# Beyond Reactive Inhibition: Unpacking the Multifaceted Nature of Motor Inhibition

**DOI:** 10.3390/brainsci13050804

**Published:** 2023-05-16

**Authors:** Giovanni Mirabella

**Affiliations:** 1 Department of Clinical and Experimental Sciences, University of Brescia, 25123 Brescia, Italy; giovanni.mirabella@unibs.it; Tel.: +39-030-3717450; 2 IRCCS Neuromed, 86077 Pozzilli, Italy

Inhibition is a pillar of cognitive control, i.e., the ability to select, regulate, and coordinate mental representations of internal states and sensory evidence to choose the most appropriate behavioral strategy, overcoming inappropriate habitual actions. About ten years ago, Braver [1] postulated that cognitive control functions through two qualitatively distinct modes of operation, namely reactive control and proactive control. The former reflects a mechanism exploited at need when internal or external salient events occur, requiring an abrupt change in behavior. Instead, the proactive mode involves a set of processes that enables subjects to maintain goals in a sustained manner before the occurrence of cognitively interfering events. Reactive and proactive control differ in terms of resource consumption. In the reactive control mode, goal representations are activated when needed. By contrast, in the proactive mode, goal representations are continuously maintained, and thus, more resources must be employed. The account of Braver [1] was meant to provide a framework of the operating mode of all cognitive functions as an ensemble without making any parcellation. However, experimental evidence showed that core executive functions, i.e., inhibition, shifting, and working memory [2,3], may operate in a reactive or proactive mode in isolation. Could these opposite views be reconciled? We begin with the example of inhibitory control. This is a multifaceted executive function where at least two domains can be distinguished: behavioral (or motor) and cognitive inhibition [4,5]. The former refers to the ability to inhibit prepotent motor responses and, thus, overt behaviors; the latter refers to the ability to inhibit interferences caused by mental or sensory stimuli to resolve response conflicts allowing the implementation of the more adaptive behavior. Both types of inhibition can operate in a reactive (at need) and a proactive (a priori) mode [6]. The question is, to what extent do pure reactive or proactive inhibitory modes exist? Alternatively, could they emerge from the cooperation between different executive functions? This last position is that held by Boag et al. [7]. Using a model-based approach, the authors showed that the performance in a working-memory decision task depends upon the interplay of cognitive functions, including reactive inhibition. However, although such interactions are likely to occur, it is possible that in certain task designs, one executive function could be more relevant than the others to allow the best performance. The review by Van den Wildenberg et al. [8] is particularly useful as it provides clear, experimental-based definitions of reactive and proactive inhibitory control. Van den Wildenberg et al. [8] define reactive inhibition as an adaptive mechanism that stops ongoing motor actions abruptly at the occurrence of external or internal events. Instead, proactive inhibition is defined as a preparatory process that modulates the outcome of the reactive inhibition by biasing its chances for success or failure in the future. The authors go further by suggesting the existence of two classes of proactive inhibitory control modes. One is based on amping-up/down reactive inhibition (e.g., I run fast on a busy road, but I increase my readiness to stop in case I see the police or an obstacle), the other is based on presetting in advance actions’ control (e.g., I drive slowly so that if I encounter an obstacle, I will be more likely to be able to stop). This taxonomy might be useful to better understand individual variability in inhibitory tasks in healthy people and patients with mental diseases.

The relationship between inhibitory control and clinical disorders characterized by poor urge control has been the object of several studies. Impulsivity has been frequently ascribed to deficient inhibitory control, and not seldom the two terms have been used interchangeably. However, evidence from behavioral studies is exceptionally mixed [5]. As nicely explained in the review by Morein-Zamir and Anholt [9], part of the reason is that impulsivity and inhibition are complex, partially overlapping constructs. Furthermore, most studies have focused just on reactive inhibition, disregarding proactive stopping, and the possible interactions between the two domains. In this regard, Mirabella [5] reviewed the studies on five neurodevelopmental disorders with impulsive conduct, i.e., Tourette Syndrome (TS), Obsessive–Compulsive Disorder (OCD), Attention-Deficit/Hyperactivity Disorder (ADHD), Autism Spectrum Disorder, and primary motor stereotypies (pMS), in which both reactive and proactive inhibition were assessed. He found that they showed a strikingly different pattern of impairments of motor inhibition. Reactive and proactive subdomains were severely compromised in OCD. ADHD and pMS showed a selective impairment of reactive inhibition, while ASD had a deficient proactive inhibition. By contrast, drug-naïve adolescent TS patients did not display impairments of either of the two inhibitory domains. Interestingly, in line with this piece of evidence, Morreale et al. [10] showed that adult TS patients did not show a reactive inhibition deficit with respect to healthy controls before or after the deep brain stimulation of the globus pallidus pars interna. Overall, this research suggests that the mechanisms underlying the inability to control urges are extremely heterogeneous and cannot be ascribed to an unspecific impairment of motor inhibition, thus going against the conceptualization that uniform inhibitory deficits represent a transdiagnostic feature of disorders with poor impulse control. Thus, future clinical research should assess both proactive and reactive inhibition in the same patients to gain a deeper insight into the neurocognitive mechanisms of such diseases. Morein-Zamir and Anholt [9] also underlined another crucial element that must be considered when investigating inhibition impairments in mental diseases, i.e., the fact that patients often experience failures of control under specific circumstances, e.g., when in certain affective states or under motivational pressure. To be maximally effective, laboratory tests should be performed in conditions as close as possible to those that, in real life, bring patients to experience inhibitory deficits.

This point brings us to another open challenge. While there are validated measures of reactive inhibition, e.g., the stop signal reaction time (SSRT), which is computed when using the stop signal task (SST, [11], however, see the paper by Soltanifar et al. [12]), or the percentage of commission errors, which is calculated when using the Go/No-go task, there is not yet a general agreement about how to investigate and measure proactive inhibition. As well-described by Van den Wildenberg et al. [8] taking the example of the SST, there are three common ways in which proactive inhibition has been studied. In the SST, there are two types of trials pseudo-randomly intermixed. In go trials, participants have to respond as quickly as possible to a go signal. Instead, in stop trials, a stop signal is presented after the go signal, but before the participants start to move. One way to assess proactive inhibition is by comparing two versions of the SST with different percentages of stop signal trials or with a simple reaction time (RT) task, where the percentage of stop signal trials is 0%. When stop signal trials are more frequent, responses on go trials are slower than when they are less frequent. In this instance, the effect of proactive inhibition is measured via the RT lengthening on the go trials in the condition with a relatively higher percentage of stop signal trials. Another way for assessing proactive inhibitory control is to look at the trial history during the SST. It has been shown that responses on go trials are prolonged if the trial was preceded by a stop signal trial, compared to when the go trial was preceded by another go trial. The third way is by presenting participants cues informing them about the probability of stop signal occurrence in the upcoming trial. The effect of proactive inhibition is quantified by computing the difference between the RTs of ‘certain’ go trials, i.e., preceded by a cue indicating that a stop signal would not occur, and the RTs of ‘uncertain’ go trials, i.e., preceded by a cue indicating that a stop signal might occur. The above described estimates of reactive and proactive inhibition have some limitations. Soltanifar et al. [12] challenged the reliability of SSRT estimation as performed by Logan [13], starting from the consideration that this method did not take into account the fact that go trial RTs are not constant but change according to the trial history, as just described above. Instead, the authors considered the after-effect of stop signal trials on the RTs of go trials and they provide a novel way to compute the SSRT. In other words, Soltanifar et al. [12] developed an algorithm to measure reactive inhibition, which takes into consideration participants’ proactive strategies for the first time.

The first two methods assessing proactive inhibition described above provide results that have been replicated several times; thus, there is no doubt about their validity. However, they are not very sensitive because they show little or no modulation, e.g., [14], thus providing limited information. By contrast, the third way of measuring proactive inhibition, being based on the use of cues, inevitably increases the load on attentional and working memory, failing to provide a pure measure of proactive inhibition. One solution to these caveats is to employ a reaching arm version of the SST and indexing proactive inhibition by comparing RT (the latency between go signal onset and the release of the home button) and movement times (MT, the latency between releasing the home button and pressing the response button) on go trials with similar movements in a RT task without stop signals [15]. Typically, healthy participants show a lengthening of the RTs and a shortening of the MTs of go trials in the SST with respect to the RT task. Such a phenomenon represents an optimization of the motor strategy in the two different contexts, and it has been named context effect [15]. In the SST, the anticipation of a stop signal’s presence induces a lengthening of RTs on go trials, which allows for better coding of the movement parameters. Conversely, the shorter RTs during simple RT trials do not allow it. Thus, the movement plan has to be completed during the execution of the motor response causing the lengthening of the MTs. This distinction between RT and MT has been shown to be a fruitful approach to defining the pathophysiology of different diseases characterized by poor impulse control [5]. Two articles in this special issue proposed alternative solutions. Benedetti et al. [16] used a mouse tracking procedure to capture the interactions between proactive and reactive inhibition during a SST versus two cued Go/No-go tasks (one with a higher proportion of stop trials than the other). By looking at the mouse movements occurring in response inhibition failures, they found that mouse trajectories could be unique (one-shot movements) or show multiple corrections (non-one-shot movements). In the SST, the number of one-shot movements was significantly higher than in both the cued Go/No-go tasks, and the RTs of inhibition failures were longer for the latter than the former. According to Benedetti et al., such a pattern reflects a higher tendency for online corrections of motor commands in the cued Go/No-go tasks than on the SST because the cues trigger a higher activation of proactive inhibition, determining a higher competition between conflicting motor tendencies (go vs. no-go). Ficarella et al. [17] used a combination of a Go/No-go task and a Simon task to measure the interplay between reactive and proactive inhibition. The go cues instructed participants about whether to prepare a response and what hand had to be used (left or right) in the go trials. The design was further complicated because responses with the left hand were requested more frequently than those with the right hand. The authors sought to find out how the information about response preparation would influence the performance in the Simon task, where a target stimulus could be presented ipsilaterally (compatible) or contralaterally (incompatible) to the effector with which participants had to respond. As expected, participants were faster and more accurate in compatible than in incompatible trials. However, by recording electromyographic (EMG) activity of hand muscles, the authors found that, although the behavioral response was correct in some trials, the EMG revealed an initially wrong activation, e.g., initial activation of the right-hand muscle followed by a correct left-hand response. Interestingly, such partial errors also occurred in compatible trials, i.e., when the target location and the effector used to respond coincide. According to the authors, these results indicate that go cues elicit motor preparation unspecifically, but they also activate reactive inhibition to allow participants to cancel potentially incorrect responses.

Most of the studies investigate movements performed with a single effector; however, in real life, we often use multiple effectors simultaneously. Thus, while the computational and neural mechanisms mediating movements initiation and inhibition made with single effectors have been widely studied, much less is known when more than one effector has to be employed to respond. The review by Jana et al. [18] nicely addresses such an issue, focusing on the coordination between eye and hand movements based on the results obtained using accumulation-to-threshold and race-to-threshold models. The authors suggest that the coordination of eye–hand movements follows two different modes according to the behavioral context demands. When the task requires a tight coupling between the two effectors, the initiation and the inhibition are controlled by a common command. However, when the coupling is weaker, separate commands control eye and hand movement execution and inhibition.

The other papers deal with the neural underpinnings of reactive and proactive inhibition. It is known that the network subserving this core cognitive function is extended, including both cortical and subcortical brain regions of both hemispheres [19]. The paper by Hu and Li [20] extended the list of regions involved, including the hippocampus. In more detail, they found that the activity of the right anterior hippocampus of healthy participants, measured via functional magnetic resonance (fMRI), is reduced with age and that such reduction correlates with the lengthening of the SSRT, suggesting that age-related decline in reactive inhibition could be due to a decreased activity of this brain region. By contrast, the activation of the right posterior hippocampus increased with age, which might explain the preserved proactive inhibitory control in older adults. Cattaneo and Parmigiani [21] sampled the role of the dorsal premotor cortex (PMd) by stimulating 18 different spots using single-pulse transcranial magnetic stimulation (TMS) while healthy participants performed a delayed response task with lips as effectors. The authors set a fixed delay period so that participants could implicitly learn the timing of the go signal presentation. In the sham condition, participants sometimes used a reactive strategy, i.e., they waited for the go signal. At other times they used a proactive strategy, i.e., they produced the motor response very early, without waiting for the go signal occurrence. The TMS stimulation over the rostral portion of PMd induced a shift from a reactive to a proactive strategy, speeding up lips’ movements. Instead, the stimulation of the caudal portion of PMd did not alter the motor strategy but still decreased the responses’ RTs. Overall, the authors suggest that, while the rostral part of PMd is a higher-order control station, the caudal portion exerts an inhibitory function on the primary motor cortex, acting as a low-order control station. Criaud et al. [22] focused on the still unclear function of the basal ganglia in regulating inhibition. As these structures are of a small size, are located in the depth of the hemispheres, and have complex functional-anatomic structures they are not easily accessible to investigations, especially in humans. Criaud et al. [22] studied the fMRI activity across several regions of interest, including the basal ganglia of the two hemispheres, while healthy participants performed a cued go-RT task and a cued Go/No-go task. By comparing the activity elicited in these two tasks, the authors showed that the basal ganglia are selectively involved in reactive vs. proactive inhibition. The left dorsal striatum, the left subthalamic nucleus, and the left globus pallidus internal segment were more active after the occurrence of the go/no-go signals than after the presentation of the go signal in the go-RT task, i.e., when participants always have to perform go-trials. Such findings suggest that those regions are involved in reactive inhibition. In a different manner, in the period between the cue and the go/no-go signals presentation, the right ventral striatum is more active in the Go/No-go task than in the go-RT task. Interestingly, during this period, the authors found a significant increase in functional connectivity between the ventral striatum and the visual cortex. They interpreted this evidence as suggesting that the ventral striatum enhances visual attention when participants are uncertain whether the subsequent trial will be a go- or a no-go trial, thus providing a plausible example of how proactive mechanisms could operate. Lehet et al. [23] completed an elegant study comparing inhibitory control proficiency of the saccadic oculomotor system in unaffected first-degree relatives of schizophrenic individuals with that of healthy people to understand familial vulnerability to schizophrenia. At the behavioral level, the unaffected relatives showed impaired reactive inhibition with respect to controls like schizophrenics. However, they showed better proactive inhibition than healthy people, which likely is compensation for the reactive deficit. Behavioral data correspond with the brain activity which fMRI measured. The authors used a region of interest (ROI) approach, circumscribing five brain spots involved in oculomotor control. They found an overall increase in activity and a different pattern of effective connectivity among the selected ROI in individuals at familial risk for schizophrenia with respect to controls. Thus, behavioral differences and specific brain activity alterations could potentially be used as a biomarker of schizophrenia diathesis. Finally, in a very detailed and rich review, Brockett and Roesch [24] described the role of the rat brain regions in reactive and proactive inhibitory control. Although the authors mainly focused on single-unit and lesion data collected in their laboratory, they constantly draw parallels with data obtained in non-human primates and humans. As a result, the reader can appreciate the commonalities and differences between these three models, i.e., rats, non-human primates, and humans, used to understand inhibition. Notably, in line with another review [19], they conclude that differently from what is traditionally thought, the neural network subserving inhibitory control encompasses much more than the frontal regions. As with other complex cognitive functions, reactive and proactive inhibition rely on the interactions of several cortical and subcortical brain regions that can be partially different according to the task demands.

## Conclusions

On the one hand, experimental studies of this rich collection of articles provide lab-based evidence supporting the existence of proactive and reactive inhibition as two separate domains of inhibitory control. On the other hand, theoretical papers underline the importance of such parcellation for understanding the performance of healthy people in different contexts and for characterizing the phenotypes of diseases with pure urge control. Having said this, it is very likely that in real life, core cognitive functions operate in concert. Future studies aimed at understanding the interplay of the core cognitive functions are warranted.
